# Supplementation with the Leucine Metabolite β-hydroxy-β-methylbutyrate (HMB) does not Improve Resistance Exercise-Induced Changes in Body Composition or Strength in Young Subjects: A Systematic Review and Meta-Analysis

**DOI:** 10.3390/nu12051523

**Published:** 2020-05-23

**Authors:** Josephine S. Jakubowski, Everson A. Nunes, Filipe J. Teixeira, Victoria Vescio, Robert W. Morton, Laura Banfield, Stuart M. Phillips

**Affiliations:** 1 Department of Kinesiology, McMaster University, Hamilton, ON L8S 4L8, Canada; vesciojs@mcmaster.ca (J.S.J.); nunese1@mcmaster.ca (E.A.N.); vvescio11@gmail.com (V.V.); mortonrw@mcmaster.ca (R.W.M.); 2 Department of Physiological Sciences, Federal University of Santa Catarina, Florianópolis, SC 88040-900, Brazil; 3 CBIOS—Universidade Lusófona’s Research Center for Biosciences and Health Technologies, Campo Grande, 1749 024 Lisboa, Portugal; ftperformancenutrition@gmail.com; 4 Health Sciences Library, McMaster University, Hamilton, ON L8S 4L8, Canada; banfie@mcmaster.ca

**Keywords:** muscle mass, resistance exercise, strength training, HMB, 1RM, hypertrophy, fat-loss

## Abstract

β-hydroxy-β-methylbutyrate (HMB) is a leucine metabolite that is purported to increase fat-free mass (FFM) gain and performance in response to resistance exercise training (RET). The aim of this systematic review and meta-analysis was to determine the efficacy of HMB supplementation in augmenting FFM and strength gains during RET in young adults. Outcomes investigated were: total body mass (TBM), FFM, fat mass (FM), total single repetition maximum (1RM), bench press (BP) 1RM, and lower body (LwB) 1RM. Databases consulted were: Medical Literature Analysis and Retrieval System Online (Medline), Excerpta Medica database (Embase), The Cumulative Index to Nursing and Allied Health Literature (CINAHL), and SportDiscus. Fourteen studies fit the inclusion criteria; however, 11 were analyzed after data extraction and funnel plot analysis exclusion. A total of 302 participants (18–45 y) were included in body mass and composition analysis, and 248 were included in the strength analysis. A significant effect was found on TBM. However, there were no significant effects for FFM, FM, or strength outcomes. We conclude that HMB produces a small effect on TBM gain, but this effect does not translate into significantly greater increases in FFM, strength or decreases in FM during periods of RET. Our findings do not support the use of HMB aiming at improvement of body composition or strength with RET.

## 1. Introduction

β-hydroxy-β-methylbutyrate (HMB) is a metabolite derived from the essential amino acid leucine [1]. Some research suggests that HMB is an anabolic compound that increases resistance exercise training-(RET)-induced gains in fat-free mass (FFM) [2,3,4,5,6,7,8,9]. Also, many studies have been conducted to examine the impact of HMB on body fat loss and muscle strength and performance-related outcomes [7,10,11,12]. Some studies using rodents [13,14] and non-exercising humans [15,16] have shown that HMB was able to modulate some aspects that might be directly or indirectly linked to skeletal muscle hypertrophy. For example, HMB has been shown to stimulate muscle protein synthesis in cells in vitro [17]. Also, in vitro [18] and rodent studies [19] have shown that HMB can decrease protein catabolism by modulating the activity of proteins involved in both protein synthesis and protein breakdown. As a nutritional supplement, HMB is available in two forms: a calcium-bound (HMB-Ca) form and a free-acid (HMB-FA) form [15,16]. It has been shown that 3 g of HMB-FA [15] and 3 g of HMB-Ca [16] acutely stimulate MPS to a similar extent in healthy young men.

Several reviews have been published citing the efficacy of HMB as a strategy to improve changes in body composition and/or performance during resistance exercise [20,21,22,23]. However, systematic reviews and meta-analyses are equivocal in support of the anabolic properties of HMB (Table 1). For example, Rowlands and Thomson [7] conducted a systematic review and meta-analysis in which they examined the HMB effects in trained and untrained subjects undergoing RET. The only significant finding of their meta-analysis was a small improvement in leg strength in untrained subjects, but no significant effects on body composition [7]. Similarly, a meta-analysis performed to test the effects of HMB on body composition and strength of trained and competitive athletes found no beneficial effects of HMB supplementation [11]. However, Silva et al., [24], examining only HMB-free acid (HMB-FA), concluded that HMB-FA ingestion for 12 weeks enhanced resistance training-induced increases in total body mass and fat-free mass and fat mass loss. Also, the authors concluded that HMB-FA ingestion promoted higher gains of strength and better performance in high-intensity assessments such as the Wingate, and vertical jump test [24]. Recently, Fernandez-Landa et al., [25] conducted a systematic review including only studies using the combination of HMB and creatine and concluded that HMB might have some potential effects on body composition. Nevertheless, the effect of creatine on body composition, well known to have a positive effect on body composition and strength gains [26], could not be ruled out as the sole reason for the effects observed separate from a potential effect of HMB. In sum, various meta-analyses provide disparate answers to the question of the effectiveness of HMB in enhancing body composition (increased lean mass and reduced fat mass) as well as strength during RET.

Here, we sought to conduct a comprehensive systematic review and meta-analysis on HMB with RET in untrained and trained persons. Based on a lack of any discernable difference between the HMB-FA [15] and HMB-Ca [16] forms of the supplement in terms of anabolic properties, despite an ostensibly higher bioavailability of the FA-form versus the Ca-form of HMB [29], we aimed to study both forms of HMB in this analysis. Our aim was a systematic review and meta-analysis to bring an updated evidence-based answer to whether or not HMB supplementation augments resistance exercise-induced gains in fat-free (i.e., lean) mass, reductions in body fat mass (FM), and increases in strength.

## 2. Materials and Methods

This systematic review and meta-analysis were performed according to the Cochrane Handbook for Systematic Reviews of Interventions [30] and is reported according to the PRISMA (Preferred Reporting Items for Systematic Reviews and Meta-Analysis) guidelines [31].

### 2.1. Eligibility Criteria

Studies were eligible for inclusion if they met the following criteria according to the PICOS (Participants, Intervention, Control, Outcome measurements, and Study design) strategy. Participants were healthy untrained or trained men or women between the age range of 18–50 years old. The intervention dose of HMB was set at 3.0 g HMB/day and study participants had to be conducting full body RET for ≥ 3 weeks (training sessions at least twice/week). Comparators were any placebo and the same resistance exercise program. Outcomes were total body mass (TBM), fat-free (i.e., lean) body mass (FFM), fat mass (FM), and 1RM strength. Study designs included only double-blinded-randomized clinical trials.

### 2.2. Systematic Search Strategy

A literature search for randomized controlled trials (RCT) investigating the effect of HMB on lean body mass, strength in adult male subjects was conducted by electronic searching of relevant literature databases. Two investigators (JSJ and LB) performed database searchers (last search date 1 April 2020) on the Medical Literature Analysis and Retrieval System Online (Medline), Excerpta Medica database (Embase), The Cumulative Index to Nursing and Allied Health Literature (CINAHL) and SportDiscus using a predetermined search strategy based on relevant keywords (see Supplementary material for the search strategy). Limits were applied to the electronic search, restricting studies to those including adults and humans, and published in the English language only. Abstracts and conference proceedings were not included in the present meta-analysis.

### 2.3. Data Extraction and Outcome Measures

Studies were carefully reviewed and searched for information regarding the study design, the RET intervention, subject characteristics, supplement information, placebo/control information, body composition outcomes and performance outcomes, and any other notable information (e.g., sources of bias/conflict of interest). Data was independently extracted from the selected papers by three investigators (JSJ, FJT, and VV). For some studies, corresponding authors were contacted and asked to provide raw data. The following outcomes were investigated: TBM (measured by any scale); FFM, and muscle mass (i.e., lean mass) if FFM was not available (measured by dual-energy X-ray absorptiometry (DXA), hydrodensitometry, or whole-body air plethysmography (BodPod^®^); and fat mass (FM; measured by DXA, hydrodensitometry and/or BodPod^®^). Performance outcomes were: one-repetition-maximum (isotonic) strength (1RM; measured by any 1RM strength test). For strength, bench press (BP) 1RM, and lower body (LwB) 1RM were extracted. LwB strength included leg press or squat exercises depending on which was used in the study.

### 2.4. Risk of Bias Analysis and Sensitivity Analysis

Risk of bias was assessed independently by two investigators (JSJ and FJT) according to the Cochrane collaboration risk-of-bias tool (Cochrane Handbook for Systematic Reviews of Interventions version 6.0) [30]. Studies were carefully reviewed for details, including randomization methods, participant allocation, and blinding of the subjects and researchers directly involved with the subjects or data analysis. Also, incomplete outcome data, selective reporting, and other sources of bias were assessed. Where disagreements between the two investigators (JSJ and FJT) were not resolved by consensus on study eligibility, data extraction, and risk-of-bias assessment, a third investigator reviewed the paper to yield a decision (EAN). Studies not reporting randomization or blinding procedures were considered high risk in the domain allocation concealment and blinding of participants and personnel. Industry-related sponsorship or authorship were considered “unclear risk of bias” and included in “other bias”. Funnel plots were generated to assess for evidence of asymmetry and potential publication bias [32]. Heterogeneity between studies was determined by I^2^, with values of <50% considered low, 50–74.9% considered moderate, and 75–100% considered high heterogeneity. Sensitivity analyses were also conducted. Studies assessed with 3 or more domains judged as high or unclear risk of bias were submitted to sensitivity analyses. These analyses were conducted for all outcomes by the “remove 1” technique. Such a procedure aimed to assess whether individual studies had a disproportionate effect on the results of the meta-analyses [33].

### 2.5. Data Syntheses and Meta-Analysis

Only pre-intervention and post-intervention outcome data were retrieved if a study had multiple time points. Where the SD for change (ΔSD) was available, it was collected alongside the pre- and post-intervention SD. Studies testing HMB-Ca and HMB-FA forms had results of both groups combined using the RevMan calculator [34] (RevMan, V.5.3. Copenhagen: The Nordic Cochrane Centre, The Cochrane Collaboration, 2014) and then compared to placebo groups. When necessary, missing ΔSD was imputed according to the instructions of the Cochrane handbook using correlation coefficients obtained from studies were standard deviation for changes were presented. This procedure was necessary for four studies during body mass and composition analysis and for nine studies for strength data. The change in mean (ΔMean) and ΔSD were calculated for each group and uploaded to Review Manager [34] (RevMan, V.5.3. Copenhagen: The Nordic Cochrane Centre, The Cochrane Collaboration, 2014). Total strength 1RM was independently calculated by combining bench press and leg press, leg extension or squat 1RM using the RevMan calculator [34] (Rev Man V.5.3. Copenhagen: The Nordic Cochrane Centre, The Cochrane Collaboration, 2014). Random-effects meta-analyses were performed in RevMan on the change in each outcome of interest. Effect sizes are presented as mean difference (MD) with means ± SD and 95% confidence intervals (CI). Test for overall effect (z score) was regarded significant when *p* ≤ 0.05.

## 3. Results

### 3.1. Included Studies

**Figure 1:**
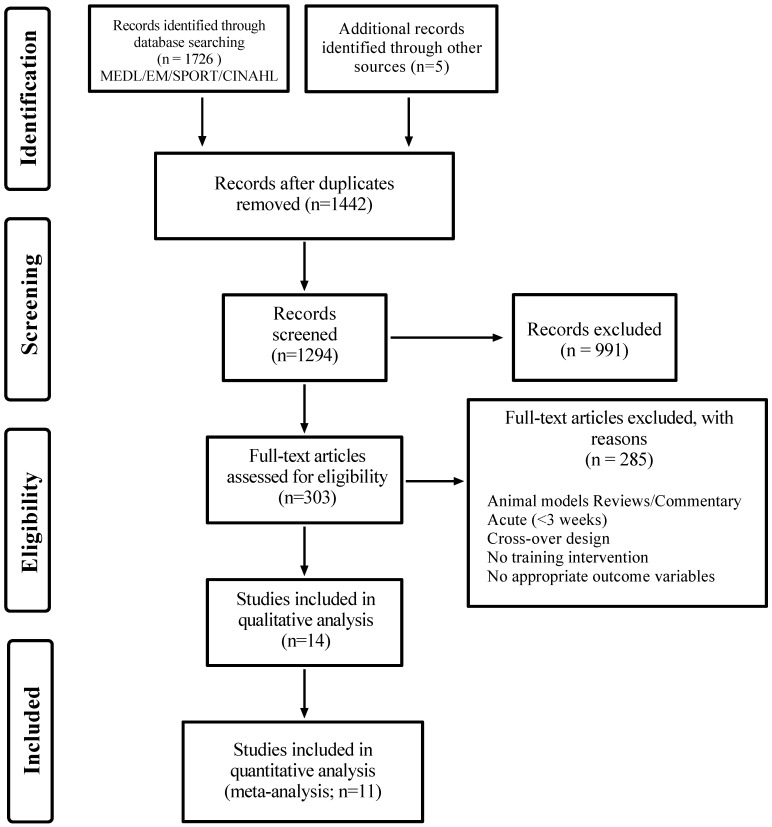
Preferred Reporting Items for Systematic Reviews and Meta-Analysis (PRISMA) flow chart. Medline: Medical Literature Analysis and Retrieval System Online; Embase: Excerpta Medica database; CINAHL: The Cumulative Index to Nursing and Allied Health Literature.

A total of 1731 results were obtained from the search strategy and additional searches during data analysis (Figure 1). After removing duplicates, in vitro studies, articles not in English, and reviews, 1294 records were available for title and abstract screening. Of these, 303 records were retrieved for full-text screening with 14 double blinded-RCTs being eligible for inclusion (Table S1).

### 3.2. Risk of Bias and Asymmetry Analysis

After selecting studies according to the inclusion and exclusion criteria, we conducted a sensitivity analysis followed by risk of bias analysis. One study was removed due to missing data [3] (Table S2). Two additional studies were excluded (Table S2) after visual funnel plots symmetry analysis (Figure S1A) [35,36]. No asymmetry was detected when considering the remaining studies [2,4,5,6,8,9,10,12,37,38,39] (Figure S1B). One study received high risk of bias classification in three domains (selection bias, performance bias and other bias) [2]. Seven studies received an unclear risk classification for detection bias (blinding of the outcome assessment), due to lack of detailed description in the respective papers (Figure S2A,B) [2,4,5,6,10].

### 3.3. Study Characteristics

After removing studies according to sensitivity analysis, 11 studies remained to be meta-analyzed (Table 2). For the analysis, one article was considered as two separate RCT because data from men and women were available and permitted the inclusion as independent data [4]. A total of 302 participants were included in body mass and composition analysis, and 248 were included in the muscle strength analysis. The average age of the participants in the studies was 27 years (18–45 years). Study duration was 7.6 ± 4.0 weeks with a training frequency varying between 2–5 days/week. Placebo was mainly carbohydrate based (e.g., rice flour, corn starch, polydextrose or microcrystalline cellulose) for most of the selected studies (Table 2).

### 3.4. Body Mass and Composition

**Figure 2:**
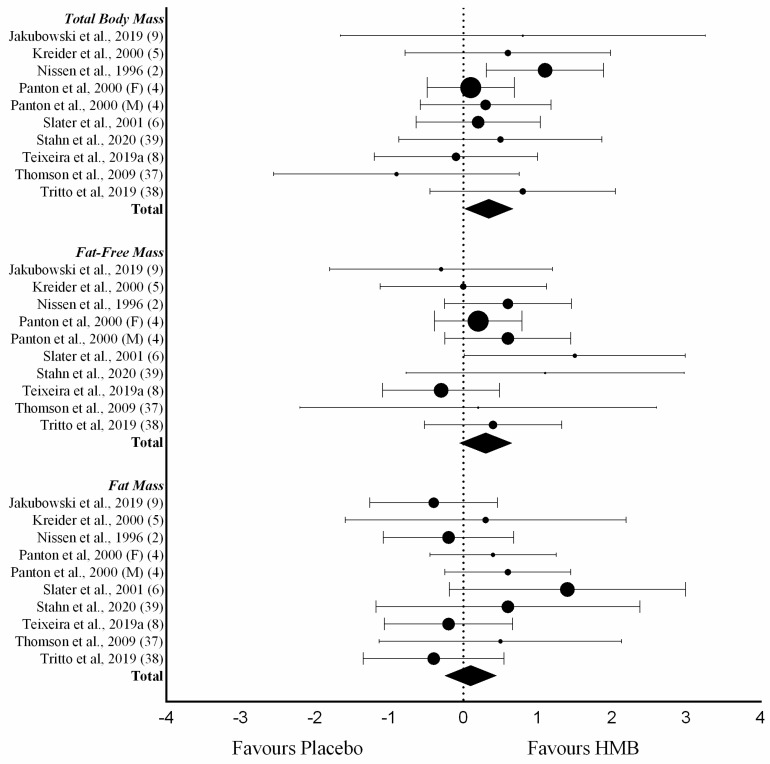
Forest plot of a random-effects meta-analysis for the effect of HMB supplementation on changes in TBM (9 studies, 10 trials), lean body mass (9 studies, 10 trials) and fat mass (10 studies, 9 trials). Results are presented as mean difference between HMB supplemented (Experimental) and Placebo group with 95% CIs. HMB, β-hydroxy-β-methylbutyrate; CIs, Confidence intervals.

Total body mass was evaluated in eleven trials. The mean TBM gain in HMB-supplemented subjects was 1.12 kg ± 1.89 kg versus 0.78 kg ± 1.31 kg gain in the placebo group. The mean difference between placebo and experimental was 0.34 kg (95% CI 0.03, 0.66, *p* < 0.05). Eleven trials measured lean body mass changes. Mean difference between supplemented and placebo groups was 0.29 kg (95% CI −0.01, 0.60, *p* = 0.06). The HMB-supplemented groups gained an average of 1.57 kg ± 1.75 kg and the placebo groups gained 1.17 kg ± 1.45 kg of FFM. Mean fat loss was equivalent in these same studies. HMB-supplemented group lost 0.73 kg ± 1.68 kg, and the placebo groups lost 0.47 kg ± 1.43 kg. The mean difference between groups was 0.10 kg (95% CI −0.42, 0.23, *p* = 0.57) (Figure 2).

### 3.5. Gains in Muscle Strength

**Figure 3:**
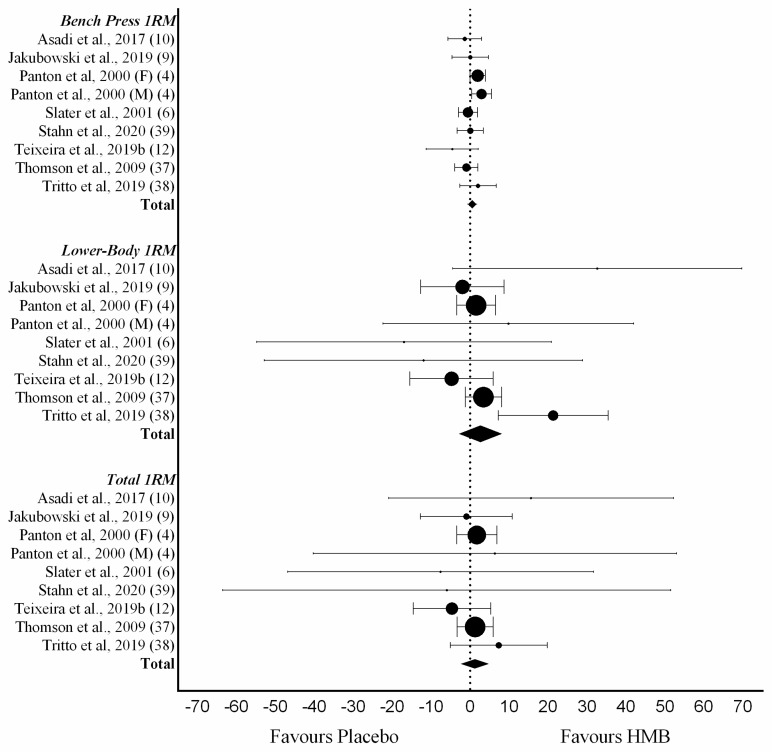
Forest plot of a random-effects meta-analysis for the effect of HMB supplementation on changes in strength. Eight studies (nine trials) were analyzed for bench press 1RM, lower body 1RM (kg) and total 1RM. Results are presented as mean difference between HMB supplemented (Experimental) and Placebo group with 95% CIs. HMB, β-hydroxy-β-methylbutyrate; CIs, Confidence intervals.

Strength was an investigated outcome in 8 of the 11 analyzed studies. In these studies, subjects that undertook RET and placebo increased their total 1RM strength by 30.6 kg ± 29.9 kg. HMB-supplemented individuals increased their total 1RM strength by 32.0 kg ± 31.4 kg. Mean difference between groups in total 1RM strength was 1.11 kg (95% CI −1.90, 4.12), which was not statistically significant (*p* = 0.47). Total 1RM strength was based on bench press and lower body 1RM data. Bench press 1RM strength gains were equivalent to 9.5 kg ± 4.6 kg in studies testing HMB supplemented subjects. In the same studies, the placebo group increased its strength by an average of 9.6 kg ± 4.9 kg. The mean difference between groups in bench press 1RM strength was not statistically significant (0.52 kg (95% CI −1.72, 1.75, *p* = 0.41)). Finally, lower body 1RM strength was also not significantly affected by HMB supplementation. The mean lower body 1RM difference between experimental and placebo groups was 2.82 kg (95% CI: −2.37, 8.00 *p* = 0.29) (Figure 3).

### 3.6. Sensitivity Analyses

All outcomes were submitted to sensitivity analysis using the “remove-1” strategy. The outcome “total body mass” was significantly influenced by the removal of 1 study. Studies were removed according to the criteria of 3 or more domains judged as high or unclear risk of bias. When the results of Nissen et al., [2] were removed from the meta-analysis, the mean difference between treatments turned out to be non-significant (0.20 kg (95% CI −0.13, 0.54, *p* = 0.20)). Therefore, the main effect of HMB supplementation in TMB, presented in Figure 2 should be interpreted with caution.

## 4. Discussion

This systematic review and meta-analysis showed that HMB ingestion does not augment RET-induced changes in body composition or strength following weeks or RET (7.6 ± 4.0 wk) in adults between the ages of 18 and 45 years old. These findings are in line with results from previous meta-analyses focusing on HMB supplementation [7,11,28]. Nevertheless, a small significant effect was observed for TBM. However, sensitivity analyses revealed that this result was strongly influenced by one study that scored as “high risk” in three domains of the risk of bias analysis [2]. This highlights the importance of a qualitative view when interpreting the results of a meta-analysis.

A notable and relevant facet of the present meta-analysis is that our conclusions were based on studies conducted in non-athletes. Although, this was not one of the main objectives of the present meta-analysis when designing inclusion and exclusion criteria, our conclusions derived mainly from studies in which untrained persons were studied (Table 2). This is important because two recent meta-analyses [11,28] showed no substantive effects of ingesting HMB when combined with resistance exercise, but on studies conducted mostly in athletes. Therefore, it seems reasonable to conclude that our data, viewed collectively with previous work [11,28], show that HMB is not a valuable nutritional supplement to be consumed by athletes or non-athletes aiming to improve resistance exercise-induced gains in FFM and strength.

HMB, as would be expected as a metabolite of leucine, can acutely activate skeletal muscle protein synthesis and the main signaling pathways leading to protein synthesis [15,16]. Increased muscle protein synthesis is a requisite for promoting a positive net muscle protein balance and increasing muscle mass as a result of RET [40]. Some studies have reported extraordinarily greater lean mass and strength gains by individuals ingesting HMB [35,36], or HMB + ATP [41] when undertaking RET. It is relevant to highlight that Kraemer et al. [35], and Wilson et al., [36] met the inclusion criteria for this meta-analysis but were excluded due to the marked asymmetry that inclusion of those studies created, as seen in the funnel plot analyses when the data were present (Supplementary Figure S1). Kraemer et al. [35], reported ~9.3 kg gain of FFM, and Wilson et al. [36] reported 7.4 kg of FFM gain over 12 weeks of resistance exercise and HMB supplementation. However, FFM gains of such magnitude are similar to those reported by the subjects submitted to resistance exercise and in the use of androgen anabolic steroids [42]. Also, these same studies [35,36] reported high FM loss (i.e., >5 kg) in the groups ingesting HMB. Nevertheless, the present meta-analysis found that average FM loss is 0.73 kg ± 1.68 kg in trials testing HMB during RET. Hence, it is reasonable, as we have stressed previously [9], to exclude studies that report seemingly unparalleled results (i.e., >7 kg in FFM) in 12 weeks of RET.

There are no studies measuring the acute protein synthetic response to a protein meal/supplement or to a resistance exercise bout in the presence of HMB. Therefore, it is not known if the acute effects of HMB on protein synthesis [15,16] are additive to the expected response to the ingestion of proteins or a resistance exercise bout, but it would seem unlikely so long as leucine was ingested at a sufficient dose [15]. Nevertheless, some studies [2,3,9,39], including one from our group [9], have tested the long-term effects of ingesting HMB together with high-quality protein on the adaptation to resistance exercise. Two of these studies [3,9], showed no significant benefit to body composition changes or strength gains caused by RET of adding HMB to protein-containing nutritional supplements, 75 g and 50 g respectively [3,9]. In addition, Shirato et al. [43] were not able to find any significant effect of adding only HMB (3 g) to a ~37 g/day of whey protein supplement on the recovery from eccentric exercise. This is relevant because one of the main statements used during the prescription of HMB is based on its potential effects on accelerating skeletal muscle recovery after resistance exercise sessions [24]. In contrast, Nissen et al. [2] showed a higher increase in lean body mass and strength when HMB was added to a nutritional supplement containing 37 g of milk protein. Also, Stahn et al. [39] showed that ingesting 3 g of HMB-Ca added to 30 g of whey protein was able to increase fat-free mass in young men. However, the placebo group in Stahn et al. [39] (ingesting 30 g whey protein only) did not actually show a significant increase in whole-body FFM. Therefore, the conclusions of that study [39] are perplexing, since it would be expected that 12 weeks of RET would result in a significant increase in whole-body FFM even in the placebo group [9,44]. Nevertheless, the fact that HMB is not effective in producing significant effects in young subjects performing resistance exercise does not exclude the possibility of potential effects in other populations or physiological states. In fact, a recent meta-analysis revealed a small significant effect for HMB supplementation on increasing muscle mass and function in a variety of clinical conditions characterized by loss of skeletal muscle mass and weakness [45]. However, older subjects performing physical activity seem not to have further benefits on physical function by ingesting HMB when compared to performing physical activity only [46]. Finally, the ingestion of HMB by subjects performing non-resistance exercise activities might also produce distinct results when compared to our conclusions since a few trials have shown significant changes in body composition and performance variables as an effect of HMB supplementation [47,48].

One important limitation of this meta-analysis is the number of studies fitting inclusion/exclusion criteria and remaining after screening by the risk of bias analysis. Eleven studies might be considered a small number when drawing final conclusions. However, this analysis is in line with some of the recently published meta-analyses studying HMB [11,28]. In addition, although there was no significant heterogeneity observed in the analysis of strength gains, the variability between RET protocols might be another limiting factor. However, 8 out of 11 selected studies used RET protocols with a training frequency of 3 days/week or more. Additionally, six studies utilized RET programs of at least 8 weeks (Table 2). Protein ingestion was also cited as a potential limiting factor influencing resistance exercise responses in HMB-supplemented individuals [28]. Holland et al. [28] postulated that HMB might not enhance RET gains in individuals already ingesting >1.6 g of protein/day. Still, only two of the seven selected studies by their meta-analysis reported daily protein ingestion of >1.6 g of protein/day and additional four studies did not report protein ingestion. Hence, in our view any statement related to such protein-dependence on daily protein ingestion is tenuous. In our meta-analysis, 6 out of 11 studies reported protein ingestions in the range of 1.9–3.3 g/kg/day, and one additional study reported daily protein ingestion of 1.45 g/kg. Also, three studies did not state the protein ingestion. Based on such data, it is not possible to perform any analysis as to what protein intake might be needed to see an effect of HMB supplementation. We would posit, however, that dietary protein, even though its impact on hypertrophy is relatively small [44], would be far more potent than a single leucine metabolite.

## 5. Conclusions

This systematic review and meta-analysis showed that HMB supplementation during RET may result in a small increase in TBM but does not result in a significant enhancement of gains in FFM or losses of FM. Thus, there is no rationale for prescription of HMB as a supplement to improve body composition caused by RET in young subjects. In addition, effects on strength were also not significant. Furthermore, risk of bias and sensitivity analysis suggest that some studies reporting significant HMB effects to optimize RET adaptations are not commonly reproducible [35,36] or have a considerable risk of bias [2]. Our results, particularly when viewed in light of other meta-analyses reaching similar conclusions [11,28], show that HMB is not an effective anabolic supplement.

## Figures and Tables

**Table 1:** Main findings of previous meta-analyses assessing effects of β-hydroxy-β-methylbutyrate (HMB) ingestion in lean body mass and strength gains in subjects submitted to a resistance exercise program.

Study	Objective	Criteria	Included Studies	Supplement	Outcome	Conclusion	Lean Mass	Strength	Effect Size	
									Lean Mass	Strength
Nissen and Sharp (2003) [27]	To quantify dietary supplements to augment lean mass and strength gains during Resistance Exercise Training (RET)	Randomized clinical trial (RCT) Duration: Full Body RET ≥ 3 wk Frequency: ≥ 2 x/wk	9 studies - Nissen et al. (1996) [2] included as 2 studies	3 g HMB-Ca	Lean Mass Total Strength	There is sufficient data to support the use of HMB to augment lean mass and strength gains with RET	HMB results in an increase of 0.28%/wk confidence interval (CI): 0.13% to 0.42%	HMB results in an increase of 1.40%/wk CI: 0.41% to 2.39%	Trivial effect size (ES): 0.15 CI: 0.06 to 0.24 *p* < 0.005	Trivial ES: 0.19 CI: 0.09–0.29 *p* < 0.01
Rowlands and Thomson (2007) [7]	To meta-analyze the effectiveness of HMB on strength, body composition, and muscle damage in trained (TR) and untrained (UT) participants during RET	RCT 8/9 Crossover 1/9 TR or UT men No criteria for duration	9 studies N = 394	1/9 HMB + drink (whey protein-carbohydrate, vitamins, minerals, glutamine, and chromium picolinate) 1/9 HMB+ glucose+ taurine+ disodium phosphate+ potassium phosphate 2/9 HMB + 50 mg of potassium phosphate	Lean Mass Strength Creatine kinase (CK)	HMB supplementation results in a small beneficial increase to overall strength in UT lifters but has a negligible effect on TR lifters. In UT participants HMB results in a small increase in lower-body strength, In both trained and untrained lifters, the effect of HMB supplementation on body composition is negligible.	Fat-free mass (FFM) increases and changes in fat mass (FM) in UT and TR lifters were negligible	UT: Small benefit Lower-body: 9.9% ± 5.9% Average strength: 6.6% ± 5.7% Negligible gains Upper-body strength: 2.1% ± 5.5% TR: All outcomes Trivial UT and TR combined: Trivial Average strength: 3.7% ± 2.4%	Trivial 3-unit increase of HMB daily dose ES: −0.07 ± 0.17	Trivial 3-unit increase of HMB daily dose ES: 0.02 ± 0.2
Sanchez-Martinez et al. (2018) [11]	Examine the effectiveness of HMB supplementation on strength and body composition in trained athletes	RCT or cross-over TR (≥ 1 RET) or competitive athletes	6 studies - Slater et al. (2001) [6] included as 4 studies - Kreider et al. (1999) [3] included as 2 studies	5/6 RCT 1/6 crossover 4/6 3 g HMB-Ca 2/6 did not specify HMB-FA/HMB-Ca 1/6 HMB + drink (proteins, carbohydrates, vitamins, minerals) 1/7 HMB+ L-Carnitine, Choline, Boron and Garcinia Cambogia	Body mass Fat-free mass Fat mass Bench Leg press	No effect of HMB on strength and body composition in competitive athletes	HMB has a negligible effect on body composition and strength in trained and competitive athletes		Body mass ES = −0.01, CI: −0.29 to 0.27 Fat free mass ES = 0.16, CI: −0.5 to 0.46	Bench press ES = 0.0, CI: −0.32 to 0.32 Leg press ES = −0.09, CI: 0.46 to 0.28
Holland et al. 2019 [28]	Determine the effects of HMB on body composition in athletes	RCT Minimum supplement period of 4 wk	Body Mass: 7 studies, N = 208 Fat Free and Fat Mass 5 studies, N = 161 and N = 128, respectively	HMB * Does not differentiate HMB-Ca vs. HMB-FA	Body Mass Fat Free Mass Fat Mass	HMB may have a small, positive impact on FFM in athletes when protein intake is suboptimal (<1.6 g/kg/day)	There was no significant effect of HMB on FFM, although the CI was skewed in favor of a small effect There was no significant effect of HMB on BM		ES = −0.30 ± 0.13; 95% CI: 20.07 to 0.68 (*p* = 0.00)	Body Mass ES = 20.02 ± 0.04; 95% CI: 20.14 to 0.10 (*p* = 0.70)

Abbreviations: CI = Confidence Interval; CK = Creatine Kinase; ES = Effect Size; FFM = Fat free mass; FM = Fat mass; RCT = randomized clinical trial; RET = Resistance Exercise Training; TR = Trained; UT = Untrained.

**Table 2:** Characteristics of the analysed studies.

Study	Country	Design	Participants			Intervention					Outcome Measure				Dietary Assessment and Protein Ingestion
			Sex	Age	Training Status	Dose or Placebo	Duration (Weeks)	Training	HMB n	Control n	Strength		Body Composition		
											Upper Body	Lower Body	Fat-free mass	Fat mass	
Asadi et al., 2017 [10]	Japan	Randomized controlled trial (RCT) Double blinded (DB)	Male(M)	21.4 ± 0.7	Not Described	3 g HMB-FA or polydextrose	6	2 x/w 3 sets of 8–12 repetitions per set (rep) at 75–85% of 1 repetition maximum (RM)	8	8	Bench press	Leg Press	---	---	3-day diet records week 0 and week 6 Protein ingestion ~1.45 g/kg/d
Jakubowski et al., 2019 [9]	Canada	RCT DB	M	22.5 ± 2.2	Trained (TR) (Recreationally trained—2x/week)	3 g HMB-Ca + 50 g Whey Protein or 50 g Whey Protein	12	3–5 x/w Phase 1: 8 w Undulating periodized resistance-training (UPRT) Phase 2: 2 w overreaching Phase 3: 2 w	13	13	Bench press	Squat	Dual X-ray absorptiometry (DXA)	DXA	3-day diet records weeks 0,8 and 12. Protein ingestion ~1.8–1.9 g/kg/d
Kreider et al. (2000) [5]	USA	RCT DB	M	20.0 ± 1.5	TR	3 g HMB-Ca, 99 g/d of glucose, 1.1 g Na_2_HPO_4_, 1.2 K_2_PO_4_ and 3 g/d of Taurine or a placebo without HMB-Ca	4	4 x/week, 1 to 3 sets of 2–8 rep, 60 to 95% of 1RM (+ 3x week of agility /sprint training)	14	14	Bench Press	Squat	DXA	DXA	4-day nutritional intake assessment day 0 and day 28. Protein ingestion 1.5–2.4 g/kg/d
Nissen et al., 1996 [2]	USA	RCT DB? (not clear)	M	19–29	Untrained (UT) (at least 3 months)	3 g HMB-Ca + MET-Rx (37 g milk protein) or MET-Rx	7	3 x/w 3 sets of 3–5 rep at 90% of 1RM.	14	14	Several upper body exercises	Several lower body exercises	Total body electrical conductivity (TOBEC)	TOBEC	No dietary assessment for study 2. Estimated protein intake ~1.8–2 g/kg/d
Panton et al., 2000 [4]	USA	RCT DB	M/Female(F)	25 ± 1.2 (M) 23 ± 0.6 (F)	TR (> 6 months experience)/UT (not training for at least 6 months)	3 g HMB-Ca or rice flour	4	3 x/w. 3–6 rep 90% 1RM.	39 (21M/18F)	36 (18M/18F)	Bench press	Leg Press (M) Leg Extension (F)	UWW	UWW	Not described
Slater et al. (2001) [6]	Australia	RCT DB	M	24.5 ± 1.7	TR	3 g HMB-Ca (Standard encapsulation vs. Time Release) or rice flour	6	2–3 x/w. 4–6 repetitions for 3–5 sets (24 to 32 sets per session)	7	7	Bench press	Leg Press	DXA	DXA	Regularly dietary logs Pre 1.7 g/kg/d Post 2.4 g/kg/d
Stahn et al. (2020) [39]	USA	RCT DB	M	22.1 ± 1.5	UT (for the past 6 months)	3 g HMB-Ca + 30 g Whey Protein (daily). +30 g carbohydrate supplement only on training days or the supplements + microcrystalline cellulose as placebo	12	4x/w upper/lower body split routine. Weeks 1–6: linear periodization. Week 7 tapering. Weeks 8–12: undulating periodization	8	7	Bench Press	Leg Press	Bioelectrical impedance (BIA)	BIA	No dietary assessment
Teixeira et al., 2019a [8]	Portugal	RCT DB	M	31.7 ± 7.6	TR (>1 year experience)	3 g HMB-Ca or 3 g HMB-FA or Mg stearate as placebo	8	3 x/w Weeks 1–3, 3–4 sets 12RM Weeks 4–6, 3–4 sets 10RM Weeks 7–8, 4 sets 8RM	20	10	---	---	DXA	DXA	3-day dietary logs weeks 0, 4 and 8. Dietary instructions to adjust energy and protein ingestion. Protein ingestion 3.0–3.3 g/kg/d
Teixeira et al., 2019b [12]	Portugal	RCT DB	M	31.7 ± 7.6	TR (>1 year experience)	3 g HMB-Ca or 3 g HMB-FA or Mg stearate as placebo	8	3 x/w Weeks 1–3, 3–4 sets 12RM Weeks 4–6, 3–4 sets 10RM Weeks 7–8, 4 sets 8RM	20	10	Bench Press	Squat	---	---	3-day dietary logs weeks 0, 4 and 8. Dietary counselling to adjust energy and protein ingestion Protein ingestion 3.0–3.3 g/kg/d
Thomson et al., 2009 [37]	New Zealand	RCT DB	M	24 ± 4	TR (>3 years experience)	3 g HMB-Ca or corn starch	9	3 x/w	13	9	Bench press	Leg extension	BIA	BIA	Diets evaluated by 3-day records at weeks 0 and 9. Protein ingestion: not stated.
Tritto et al., 2019 [38]	Brazil	RCT DB	M	25.3 ± 3.7	TR (>6 months experience)	3 g HMB-Ca or 3 g HMB-FA or corn starch	12	2 x /w 3–4 sets 8–10 RM	29	15	Bench press	Leg Press	DXA	DXA	Diets evaluated by 3-day records at weeks 0 and 12. Protein ingestion: 1.9–2.1 g/kg/d.

M: Male; F: Female; BIA: Bioelectrical impedance; DB: Double blinded; DXA: Dual X-ray absorptiometry; FFM: Fat-free mass; FM: Fat mass; RCT: Randomized controlled trial; rep: repetitions per set; RM; Repetition maximum; TOBEC: Total body electrical conductivity; TR: Trained; UPRT: Undulating periodized resistance-training; UT: Untrained; UWW: Underwater weighting.

## Supplementary Materials

The following are available online at https://www.mdpi.com/2072-6643/12/5/1523/s1: Figure S1: Funnel plots showing relation between mean differences (MD) in x axis and standard errors (SE) for mean differences. Figure S2: Risk of bias summary for selected studies. Table S1: Characteristics of the studies eligible for inclusion. Table S2: List of studies removed from the meta-analysis after data collection and asymmetry with reason for exclusion. File S1—Search Strategy.
