# Supplementary material for: Supplementation with the Leucine Metabolite β-hydroxy-β-methylbutyrate (HMB) does not Improve Resistance Exercise-Induced Changes in Body Composition or Strength in Young Subjects: A Systematic Review and Meta-Analysis
Source: Nutrients. 2020 May 23;12(5):1523. doi: 10.3390/nu12051523 (PMC7285233; doi:10.3390/nu12051523)
Supplement: Supplementary file 1 [file nutrients-12-01523-s001.pdf]

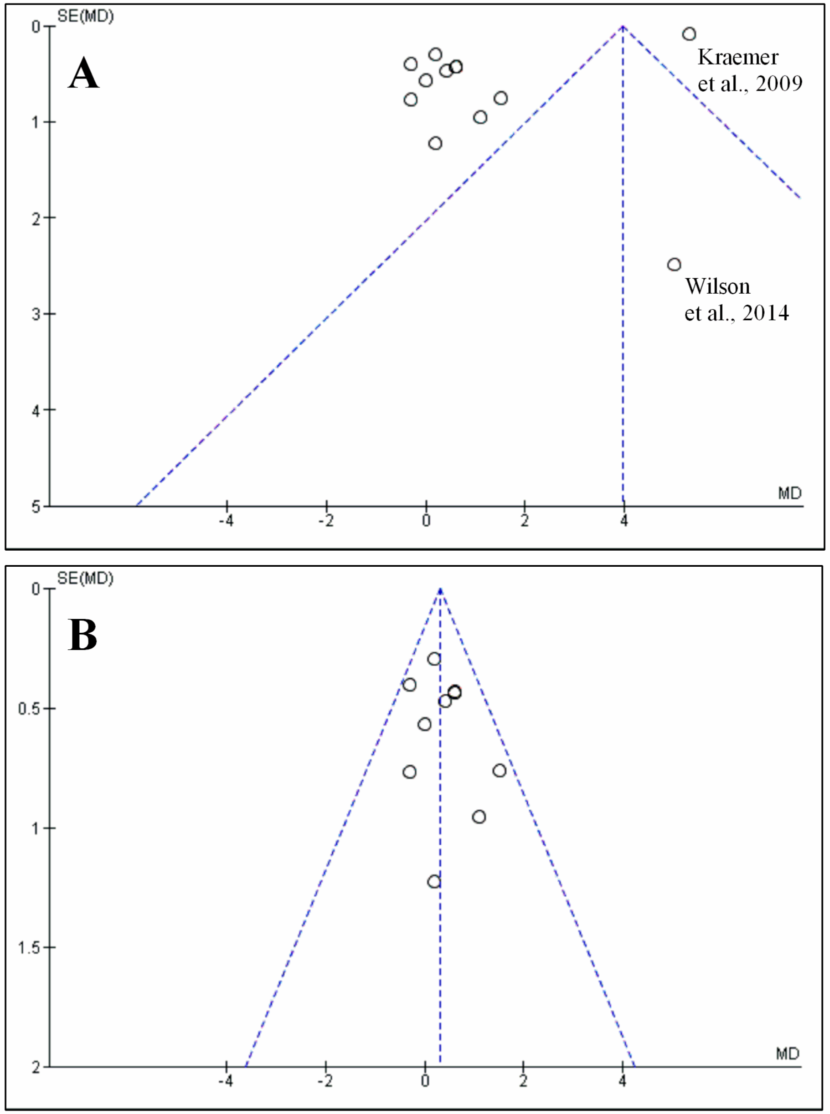

Figure S1: Funnel plots showing relation between mean differences (MD) in x axis and standard errors (SE) for mean differences.

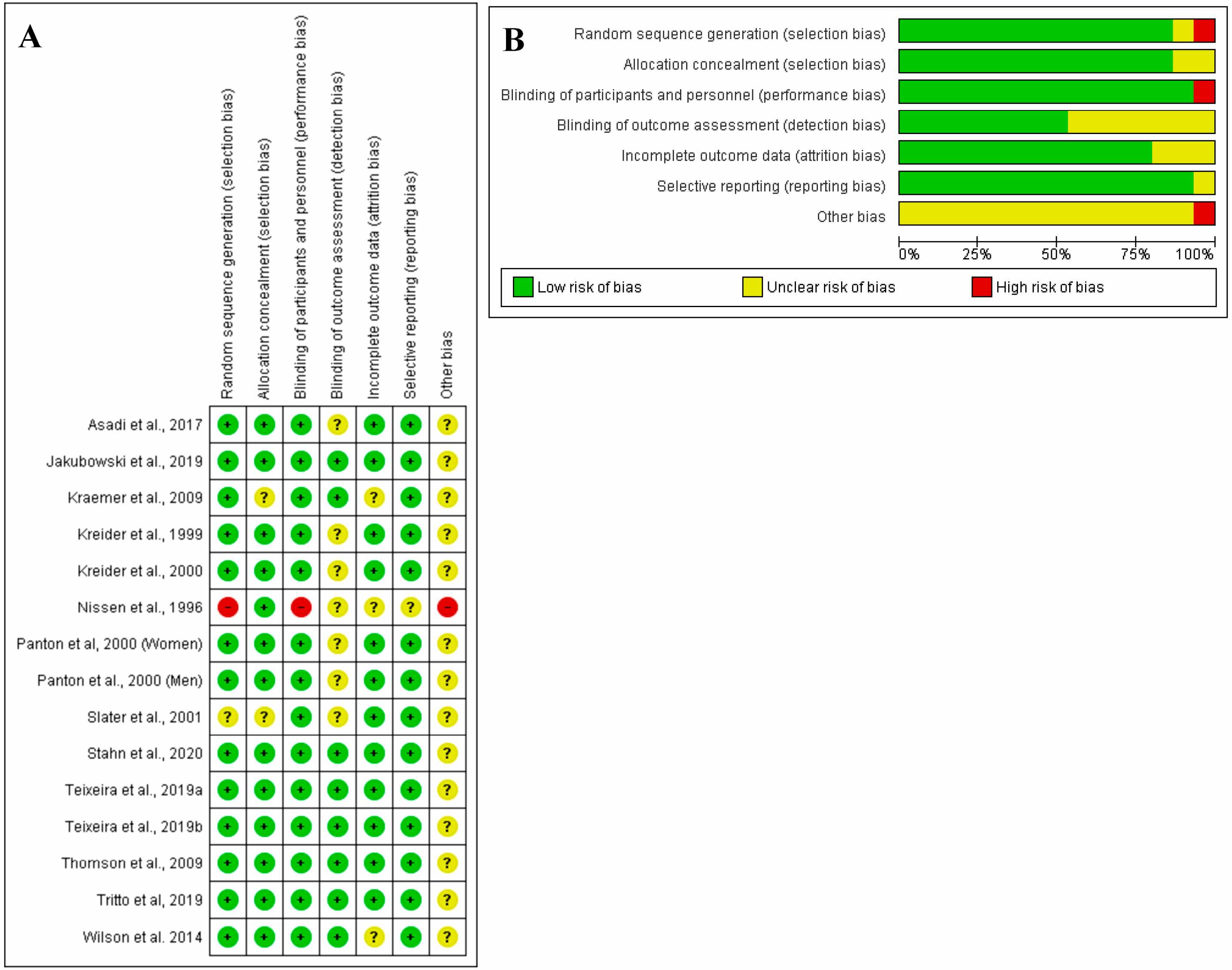

Figure S2: Risk of bias summary for selected studies.

Table S1 **- Characteristics of the studies eligible for inclusion**

Study	Country	Design	Participants			Intervention					Outcome Measure			
			Sex	Age	Training Status	Dose	Duration (Weeks)	Training	HMB n	Control n	Strength		Body composition	
											Upper Body	Lower Body	FFM	FM
Asadi et al., 2017[10]	Japan	RCT DB	M	21.4±0.7	Not Described	3g HMB-FA	6	2x /w 3 sets of 8–12 rep at 75–85% of 1RM	8	8	Bench press	Leg Press	---	---
Jakubowski et al., 2019[9]	Canada	RCT DB	M	22.5±2.2	TR	3g HMB-Ca + 50g Whey Protein	12	3-5x /w Phase 1: 8w UPRT Phase 2: 2w overreaching Phase 3: 2w	13	13	Bench press	Squat	DXA	DXA
Kraemer et al (2009)[35]	USA	RCT DB	M	22.9 ± 2.8	TR	3 g HMB-Ca 10g Glycine, 11.5g Alanine, 1.5g Glutamic Acid, 1.5 g Serine	12	3x /w UPRT	8	9	Bench press	Squat	DXA	DXA
Kreider et al (1999)[3]	USA	RCT DB	M	25.1 ± 1	TR	3 g HMB-CA Drink: 81g CHO,75g PRO, 3g FAT	4	6.9 ± 0.5 hr/w	?	?	Bench press	Leg Press	DXA	DXA
Kreider et al (2000)[5]	USA	RCT DB	M	20.0±1.5	TR	3g HMB-Ca, 99 g/d of glucose, and 3 g/d of taurine.	4	4x/w , 1 to 3 sets of 2-8 rep, 60 to 95% of 1 RM (+ 3x week of agility /sprint training)	14	14	Bench Press	Squat	DXA	DXA

Nissen et al., 1996[2]	USA	RCT DB?	M	19-29	UT (at least 3 months)	3 g HMB-Ca +MET-Rx TM (37g milk protein)	7	3x /w 3 sets of 3–5 rep at 90% of 1 RM.	14	14	Several upper body exercises	Several lower body exercises	TOBEC	TOBEC
Panton et al (2000)[4]	USA	RCT DB	M/F	25 ± 1.2 (M) 23 ± 0.6 (F)	TR	3 g HMB-Ca	4	3 x/w. 3-6 rep 90% 1RM.	39 (21M/18F)	36 (18M/18F)	Bench press	Leg Press (M) Leg Extension (F)	UWW	UWW
Slater et al (2001)[6]	Australia	RCT DB	M	24.5±1.7	TR	3 g HMB-Ca (Standard encapsulation vs. Time Release)	6	2-3x /w. 4-6 repetitions for 3-5 sets (24 to 32 sets per session)	7	7	Bench press	Leg Press	DXA	DXA
Stahn et al (2020)[39]	USA	RCT DB	M	22.1±1.5	UT (for the past 6 months)	3 g HMB-Ca + 30g Whey Protein (daily). +30g carbohydrate supplement only on training days.	12	4x /w upper/lower body split routine. Weeks 1-6: linear periodization. Week 7 tapering. Weeks 8-12: undulating periodization	8	7	Bench Press	Leg Press	BIA	BIA
Teixeira et al., 2019a[8]	Portugal	RCT DB	M	31.7±7.6	TR	3g HMB-Ca or 3g HMB-FA	8	3x /w Weeks 1-3, 3-4 sets 12RM Weeks 4-6, 3-4 sets 10RM Weeks 7-8, 4 sets 8RM	20	10	---	---	DXA	DXA

Teixeira et al., 2019b[12]	Portugal	RCT DB	M	31.7±7.6	TR	3g HMB-Ca or 3g HMB-FA	8	3x /w Weeks 1-3, 3-4 sets 12RM Weeks 4-6, 3-4 sets 10RM Weeks 7-8, 4 sets 8RM	20	10	Bench Press	Squat	---	---
Thomson et al (2009)[37]	New Zealand	RCT DB	M	24 ± 4	TR	3 g HMB-Ca	9	3x /w	13	9	Bench press	Leg extension	BIA	BIA
Tritto et al, 2019[38]	Brazil	RCT DB	M	25.3±3.7	TR	3g HMB-Ca or 3g HMB-FA	12	2x /w 3-4 sets 8-10RM	29	15	Bench press	Leg Press	DXA	DXA
Wilson et al., 2014[36]	USA	RCT DB	M	21.6±0.5	TR	3g HMB-FA	12	3-5x /w Phase 1: 8w UPRT Phase 2: 2w overreaching Phase 3: 2w	11	9	Bench Press	Squat	DXA	DXA

BIA: bioelectrical impedance; CHO: Carbohydrates; DB: Double blinded; DXA: dual x-ray absorptiometry; FAT: Lipids; FFM: Free fat mass; FM: Fat mass; PRO: Protein; RCT: Randomized controlled trial; rep: repetitions per set; RM; Repetition maximum; TOBEC: Total body electrical conductivity; TR: Trained; UPRT: undulating periodized resistance-training; UT: Untrained; UWW: Under water weighting

Table S2 **– List of studies removed from the meta-analysis after data collection and asymmetry with reason for exclusion**

**Study**	**Reason for analysis exclusion**
Kraemer et al., 2009[35]	excluded based on funnel plot analysis of lean body mass data
Kreider et al., 1999[3]	missing data for performing meta-analysis, subjects per group
Wilson et al., 2014[36]	excluded based on funnel plot analysis of lean body mass data

Supplementary information – Search Strategy

Database: OVID Medline Epub Ahead of Print, In-Process & Other Non-Indexed Citations, Ovid MEDLINE(R) Daily and Ovid MEDLINE(R) 1946 to Present
Search Strategy:
----
beta-hydroxy, beta-methylbutyrate
beta-hydroxy-beta-methylbutyrate
beta-hydroxy beta-methylbutyrate
beta-hydroxy-beta methylbutyrate
HMB.tw,kf
HMB-Ca.tw,kf
HMB-Fa.tw,kf
((calcium or free acid) adj2 beta-hydroxy-beta-methylbutyrate).tw,kf
(beta-hydroxy-beta-methylbutyrate adj2 supplem*).tw,kf

Skeletal Muscle
Body composition
((lean or muscle) adj2 mass).tw,kf
Musc* adj2 Hypertrophy

Resistance Training
((resistance) adj2 (exercise or training)).tw,kf
Muscle strength

Database: OVID Medline Epub Ahead of Print, In-Process & Other Non-Indexed Citations, Ovid MEDLINE(R) Daily and Ovid MEDLINE(R) 1946 to Present
Search Strategy:
----
1 beta-hydroxy, beta-methylbutyrate.mp. (336)
2 hmb.mp. (2855)
3 HMB-Ca.mp. (12)
4 HMB-Fa.mp. (26)
5 1 or 2 or 3 or 4 (2917)
6 exp Muscle, Skeletal/ (263893)
7 muscle*.mp. (917658)
8 exp Muscle Strength/ (19245)
9 ((musc* or hand or grip) adj2 strength).mp. (55981)

10 Creatine Kinase/ (24181)
11 creatine kinase.mp. (37126)
12 Resistance Training/ (8337)
13 ((strength) adj2 training).mp. (5715)
14 Hypertrophy/ (22822)
15 muscular.mp. (125396)
16 Inflammation/ (153552)
17 inflam*.mp. (1030839)
18 exp Muscular Atrophy/ (14667)
19 sarcopenia.mp. (8626)
20 atroph*.mp. (137154)
21 or/6-20 (2129243)
22 5 and 21 (810)
23 remove duplicates from 22 (803)
24 animals/ not (humans/ and animals/) (4651948)
25 23 not 24 (721)
26 limit 25 to English language (667)

Database: Embase <1974 to 2020 April 02>
Search Strategy:
----
1 beta-hydroxy, beta-methylbutyrate.mp. (437)
2 HMB.mp. (5163)
3 HMB-Ca.mp. (8)
4 HMB-Fa.mp. (21)
5 1 or 2 or 3 or 4 (5266)
6 exp skeletal muscle/ (335102)
7 muscle*.mp. (1534153)
8 muscle strength/ (61491)
9 ((musc* or hand or grip) adj2 strength).mp. (93810)
10 Creatine kinase.mp. or creatine kinase/ (68995)
11 muscle strength/ or muscle hypertrophy/ or resistance training/ (78812)
12 ((resistance or strength) adj2 training).mp. (24119)
13 hypertrophy/ (28416)
14 muscular.mp. (123936)
15 inflammation/ (430088)
16 inflam*.mp. (1499389)
17 muscle atrophy/ (31358)

18 sarcopenia.mp. (14170)
19 atroph*.mp. (225839)
20 musculoskeletal system inflammation/ (164)
21 or/6-20 (3213445)
22 5 and 21 (1489)
23 remove duplicates from 22 (1473)
24 animals/ not (humans/ and animals/) (955029)
25 23 not 24 (1452)
26 limit 25 to english language (1370)
